# Supplementary material for: Sterol O-Acyltransferase Inhibition Ameliorates High-Fat Diet-Induced Renal Fibrosis and Tertiary Lymphoid Tissue Maturation after Ischemic Reperfusion Injury
Source: Int J Mol Sci. 2022 Dec 7;23(24):15465. doi: 10.3390/ijms232415465 (PMC9779122; doi:10.3390/ijms232415465)
Supplement: Supplementary file 1 [file ijms-23-15465-s001.zip › ijms-2011311-supplementary.pdf]

Sterol O-acyltransferase inhibition ameliorates high-fat diet-induced renal fibrosis and tertiary lymphoid tissue maturation after ischemic reperfusion injury

Supplementary figures and tables.

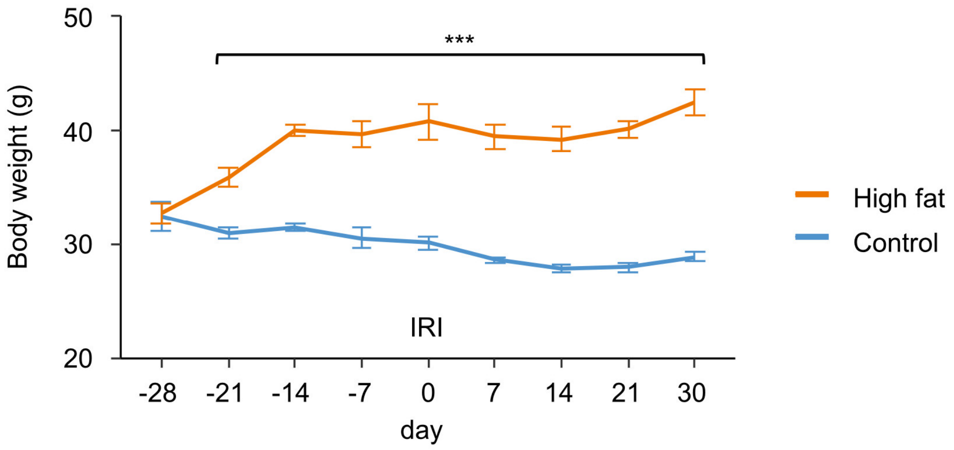

Figure S1. Body weight of mice fed a control diet or high-fat diet. Related to Figure 1. Body weight of mice fed a control diet or high-fat diet (HFD; control n = 6, HFD n = 4). Eight-month-old female mice were pretreated with the diet before 28 days of ischemic reperfusion injury (IRI) through 30 days after 35 min IRI. Values are mean ± S.E.M. Data were analyzed using student's t-test, $^{***}P < 0.001$.

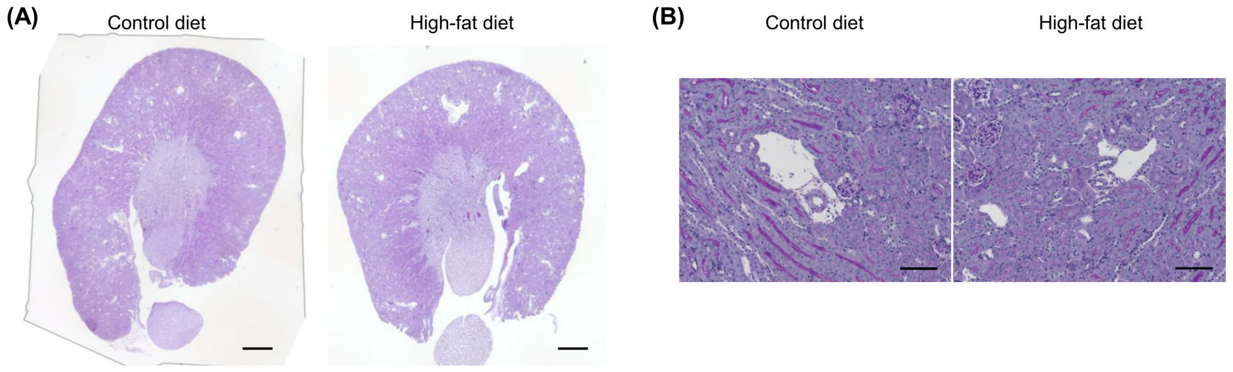

Figure S2. In the non-injured kidneys, there were no TLTs or signs of injury in both control and HFD-fed mice. Related to Figure 1. Wild-type 8-month-old female mice fed a control or HFD from 28 days prior to unilateral IRI, and diet was fed for 30 days after IRI as described in Figure 1. Non-injured sides of the kidneys were stained with periodic acid-Schiff staining. Scale bars: (A) 500 μm, (B) 100 μm.

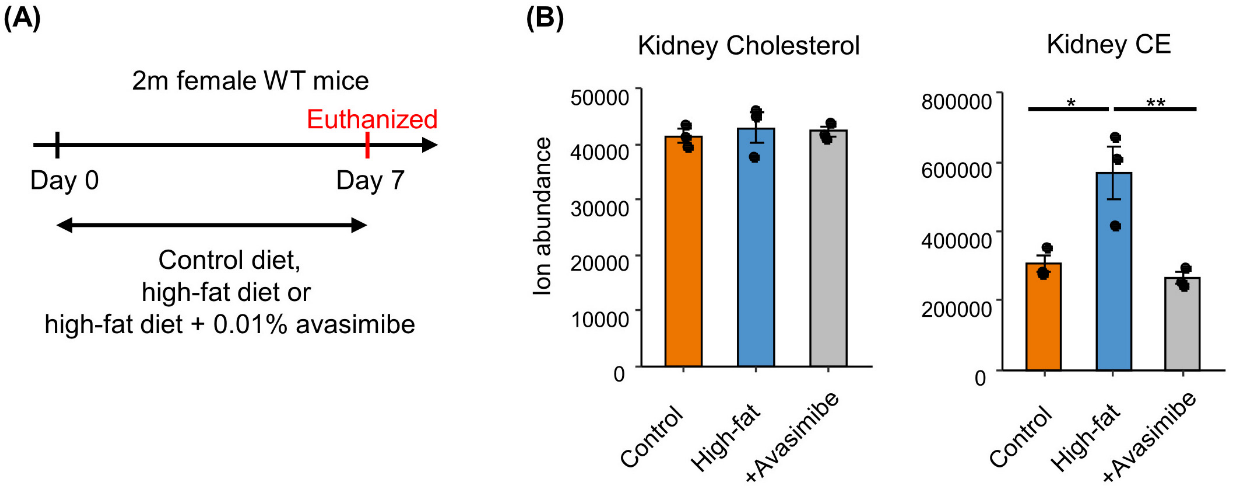

Figure S3. Comparison of kidney cholesterol and cholesteryl ester (CE) levels after different diets. Related to Figure 4. (A) Experimental protocol comparing kidney cholesterol and cholesteryl ester (CE) level after different diets. Two-month-old female mice were administered control, HFD, or HFD + 0.01% avasimibe for 7 days and kidneys were analyzed. **(B)** Kidney cholesterol and CE level after each diet detected by liquid chromatography-tandem mass spectrometry. Values are mean ± S.E.M. Data were analyzed using Tukey's HSD test, *$P < 0.05$, ** $P < 0.01$.

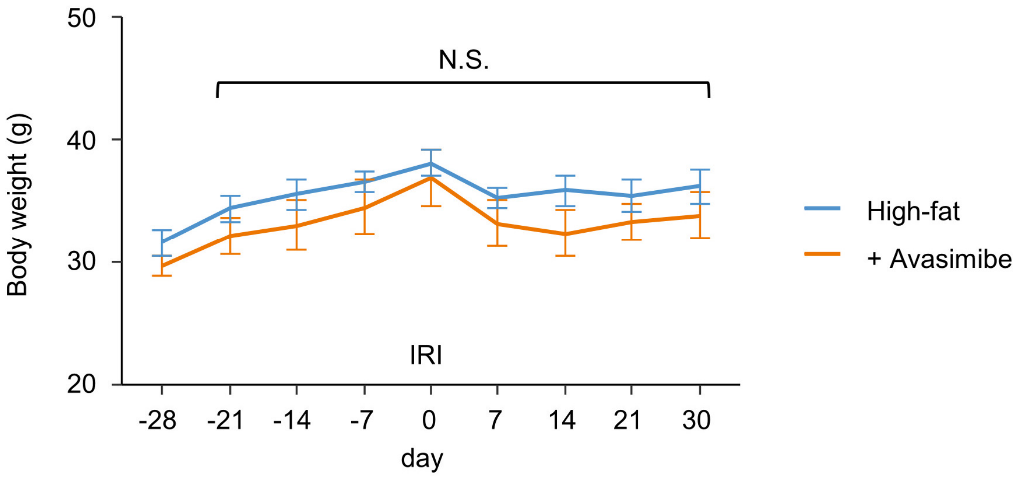

Figure S4. Body weight of mice fed a HFD or HFD + avasimibe. Related to Figure 4. Body weight of mice fed a HFD or HFD + avasimibe (HFD n = 5, HFD + avasimibe n = 7). Eight-month-old female mice were pretreated with the diet before 28 days of IRI through 30 days after 35 min IRI. Values are mean ± S.E.M. Data were analyzed using student's t-test.

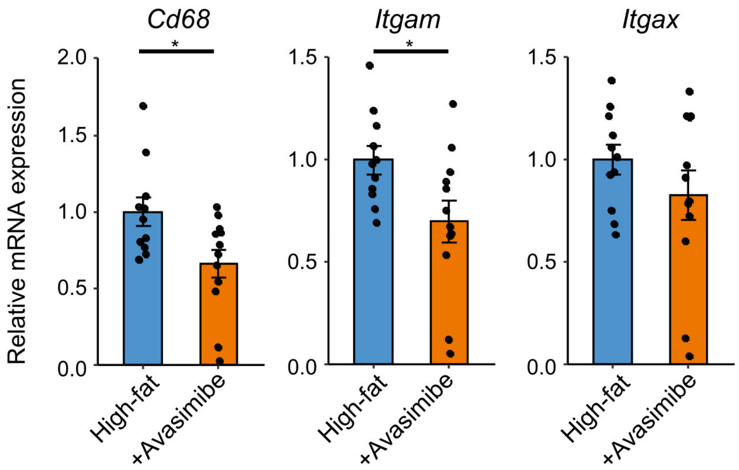

Figure S5. Relative mRNA expression of monocyte/macrophage marker comparing kidneys under HFD or HFD + avasimibe. Related to Figure 4. Relative mRNA expression of monocyte/macrophage marker comparing kidneys under HFD or HFD + 0.01% avasimibe. Eight-month-old female mice were pretreated with the diet before 28 days of IRI through 30 days after 35 min IRI (HFD n = 11, HFD + avasimibe n = 12). Data are normalized with *Gapdh* and presented as fold change relative to HFD-fed kidneys. Values are mean ± S.E.M. Data were analyzed using student's t-test, * $P < 0.05$.

Supplementary tables

Table S1. Cholesterol and cholesteryl ester (CE) levels of young (2M) and aged (12M) mice 45 days with or without ischemic reperfusion injury (IRI). Levels of aged and young mice after IRI were compared. Cells in the fold change column are colored red if the level of metabolite increased in 12M kidney with IRI kidney compared to 2M kidney with IRI. Cells in the *P*-value column colored red if $P < 0.05$ and yellow if $P < 0.1$. **Related to Figure 2.**

Metabolite	Average 2M	Average 2M IRI	Average 12M	Average 12M IRI	Fold change 12M IRI/2M IRI	*P*-value (student's *t* test) 2M IRI vs 12M IRI
CE 16:0	7150.792	9554.521	8570.328	17430.65	1.824	0.056
CE 16:1	12990.1	9362.229	16884.63	23841.33	2.547	0.070
CE 17:1	1221.896	941.5208	1308.672	1683.979	1.789	0.075
CE 18:0	1322.833	3878.646	1364.031	5469.354	1.410	0.439
CE 18:1	34607.1	25034.33	33574.64	58994.02	2.357	0.027
CE 18:2	118836.1	126581.6	146814.8	199174	1.573	0.047
CE 18:3	4812	4198.104	5876.516	6666.271	1.588	0.042
CE 20:0	108.875	1387.25	92.5625	1082.792	0.781	0.720
CE 20:1	554	2761.646	647.25	6024.479	2.181	0.072
CE 20:2	431.8542	2910.958	462.5156	5878.292	2.019	0.107
CE 20:3	3906.375	7245.208	5719.484	13943.38	1.924	0.089
CE 20:4	42910.52	80384.67	90791.31	141298.6	1.758	0.036
CE 20:5	15279.69	17571.17	29544.47	46790.08	2.663	0.014
CE 22:1	185.8542	1041.875	199.2344	1294.25	1.242	0.690
CE 22:3	61.9375	1044.729	60.20313	1105	1.058	0.930
CE 22:6	25051.48	37446.54	25095.64	118462.2	3.164	0.010
CE 24:1	177.5	971.5	115.0156	2050.396	2.111	0.054
CE 24:4	57.72917	905.1875	18.71875	1445.958	1.597	0.276
Cholesterol	5468.5	4083.708	5266.703	4030.146	0.987	0.917

Table S2. Levels of ether-linked phosphatidylcholine (PC O) in young (2M) and aged (12M) mice 45 days with or without 45 min ischemic reperfusion injury (IRI). Levels of aged and young mice after IRI were compared. Cells in the fold change column are colored red if the level of metabolite increased in 12M kidney with IRI kidney compared to 2M kidney with IRI. Cells in the *P*-value column colored red if $P < 0.05$ and yellow if $P < 0.1$. **Related to Figure 2.**

Metabolite	Average<br>2M	Average<br>2M IRI	Average<br>12M	Average<br>12M IRI	Fold change<br>12M IRI/2M IRI	*P*-value (student's *t* test)<br>2M IRI vs 12M IRI
PC O-30:0 | PC O-14:0_16:0	2340.917	3415.729	3603.188	8938.104	2.617	0.022
PC O-31:0 | PC O-16:0_15:0	702.0417	1012.271	804.3281	2159.5	2.133	0.122
PC O-32:0 | PC O-16:0_16:0	29351.17	31761.35	33902.39	60381.13	1.901	0.041
PC O-32:1 | PC O-16:0_16:1	6223.354	7709.75	10273.8	18714.71	2.427	0.035
PC O-32:1 | PC O-16:1_16:0	2356.854	4443.729	2547.078	6353.167	1.430	0.069
PC O-33:1 | PC O-21:1_12:0	539.8542	1222.563	518.4844	1741.771	1.425	0.025
PC O-34:0 | PC O-18:0_16:0	11001.31	7438.917	9668.641	8747.563	1.176	0.389
PC O-34:1 | PC O-18:1_16:0	103864.9	66911.08	101466.8	89290.42	1.334	0.149
PC O-34:2 | PC O-16:0_18:2	22468.19	18348.92	21848.95	18404	1.003	0.978
PC O-34:2 | PC O-16:1_18:1	8837.979	5663.771	8592.5	3968.979	0.701	0.009
PC O-34:3 | PC O-16:1_18:2	6018.354	4705.75	5217.5	2809.792	0.597	0.011
PC O-34:4 | PC O-14:0_20:4	1757.938	2460.625	3261.031	3084.771	1.254	0.338
PC O-36:0 | PC O-20:0_16:0	944.3542	1480.188	943.6094	2261.229	1.528	0.103
PC O-36:1 | PC O-18:0_18:1	1784.729	1482.958	1655.141	2133.854	1.439	0.123
PC O-36:2 | PC O-18:1_18:1	3591.729	3388.771	3731.547	4020.271	1.186	0.281
PC O-36:3 | PC O-18:1_18:2	6903.083	5393.292	6501.922	7070.375	1.311	0.134
PC O-36:4 | PC O-16:0_20:4	34257.35	48958.04	47635.52	63636.79	1.300	0.101
PC O-36:5 | PC O-16:0_20:5	5591.271	3949	5514.172	5971.333	1.512	0.057
PC O-36:5 | PC O-16:1_20:4	30361.98	33092.27	33055.83	22948.85	0.693	0.026
PC O-36:6 | PC O-14:0_22:6	51464.13	37756.27	93655.83	29278.35	0.775	0.171
PC O-36:6 | PC O-16:1_20:5	3925.646	2313.042	4625.234	2302.625	0.995	0.961
PC O-37:6 | PC O-15:0_22:6	18991.6	12904.69	19891.06	9401.25	0.729	0.101
PC O-38:4 | PC O-16:0_22:4	763.6042	2230.917	1055.438	3821.875	1.713	0.017
PC O-38:4 | PC O-18:0_20:4	2573.833	3491.979	4077.859	6006.458	1.720	0.011
PC O-38:5 | PC O-16:0_22:5	30431.52	28230.38	36658.92	41279.44	1.462	0.010
PC O-38:5 | PC O-18:1_20:4	5845.333	7119.229	6801	6732.208	0.946	0.724
PC O-38:6 | PC O-16:0_22:6	1266383	901492.8	1347561	688373.8	0.764	0.073
PC O-38:7 | PC O-16:1_22:6	13655.25	9917.583	17448.66	8929.458	0.900	0.401
PC O-38:7 | PC O-18:3_20:4	8924.979	7392.688	9952.688	5964.333	0.807	0.166
PC O-38:8 | PC O-16:2_22:6	3384.083	2197.729	5126.922	1725.208	0.785	0.365
PC O-39:4 | PC O-19:0_20:4	10936.5	9787.375	10797.77	8383.896	0.857	0.386

PC O-39:6 ∣ PC O-17:0_22:6	6219.729	3041.563	6057.563	2213.813	0.728	0.112
PC O-40:4 ∣ PC O-16:0_24:4	487.7917	1353.167	572.0781	2075.875	1.534	0.114
PC O-40:6 ∣ PC O-18:0_22:6	73439.5	31293.17	66112.95	23638.4	0.755	0.121
PC O-40:7 ∣ PC O-18:1_22:6	91917.5	46524.67	88341.66	39458.4	0.848	0.239
PC O-40:8 ∣ PC O-18:2_22:6	39139.29	17289.92	37437.45	9230.438	0.534	0.001
PC O-40:9 ∣ PC O-18:3_22:6	27133.83	12742	31465.56	7871.25	0.618	0.024
PC O-42:10 ∣ PC O-22:6_20:4	648.2083	1037.958	1081.109	1541.792	1.485	0.078
PC O-42:6 ∣ PC O-20:0_22:6	875.3958	422.6042	949.1406	379.7083	0.898	0.562
PC O-42:9 ∣ PC O-20:3_22:6	231.0417	486.5625	241.0781	1325	2.723	0.008
PC O-48:7 ∣ PC O-26:1_22:6	3387.375	2229.5	2032.047	1757.167	0.788	0.137
PC O-50:8 ∣ PC O-28:2_22:6	1655.188	672.0833	1000.125	838.875	1.248	0.181

Table S3. Primers used in RT-qPCR analysis, related to Method.

Gene	Sequence (5'-3')	
	Forward	Reverse
Gapdh	ACGGCAAATTCAACGGCACAGTCA	TGGGGGCATCGGCAGAAGG
Cxcl13	CGTGCCAAATGGTTACAAAGATT	GTGGCTTCAGGCAGCTCTTC
Ccl19	CCTGGGAACATCGTGAAAGC	TGGAGGTGCACAGAGCTGATA
Ifng	CTCATGGCTGTTTCTGGCTGTTAC	TTTCTTCCACATCTATGCCACTTG
Tnfa	GATCGGTCCCCAAAGGGATG	CACTTGGTGGTTTGCTACGAC
Soat1	TCGATGACTTTGTGACCAACC	TCCACTTCAAACAGCTCGTCT
Soat2	CCTGTCTTTGCCAACATGAGCC	AGGCGAAGAAGATGAGGAGCAG
Nceh1	CGGTATTTCTGGAGACAGTGCTG	GGTGTGTTGAAGTCCAAAGCCTG
Cd68	CTTCCCACAGGCAGCACAG	AATGATGAGAGGCAGCAAGAGG
Itgam	ATGGACGCTGATGGCAATACC	TCCCCATTCACGTCTCCCA
Itgax	CTGGATAGCCTTTCTTCTGCTG	GCACACTGTGTCCGAACTCA

Supplementary data

Data S1. Result of untargeted lipidomic analysis of young (2-month-old) and aged(12-month-old) kidneys with or without injury, Related to Figure 2. Data S1A represents all molecules annotated, and Data S1B represents hierarchical clustering of molecules significantly changed among any of the groups, elucidated with one-way analysis of variance test ($P < 0.05$).

Data S2. Result of untargeted analysis of mice fed different diets. Related to Figure 4. Annotated molecules in untargeted analysis of 2-month-old female mice kidney fed a control diet (ctrl), high-fat diet (HFD), or HFD added 0.01% avasimibe (HFD + Avs) for 1 week are shown.
